# Vexed Questions in Medical Ethics

**Published:** 1858-02

**Authors:** 


### EDITORIAL.

### VEXED QUESTIONS IN MEDICAL ETHICS.

Under this caption we find in the January number of the *Charleston Medical Journal*, an article from the pen of Dr. S. H. Dickson, the highly esteemed Professor of Institutes and

Practice of Medicine in the Medical College of the State of South Carolina. The writer refers to the questions involved in the conduct of Dr. Uhl in the case of Mrs. Cunningham, of New York; the appointment of Dr. McClintock to the Hospital in Philadelphia; and the proposed appointment of homœopaths to places in public Hospitals, or as professors in Medical Schools. With the views expressed by Dr. Dickson in relation to the first two topics we can cordially agree. What he says in relation to the third topic is as follows:

A very different question, the nature of which has been by some hasty thinkers confounded with the above, is presented in the appointment to office of heterodox or schismatic practitioners or teachers among those of accredited or orthodox views. Thus, in at least two of our great cities, it is proposed by the commissioners in charge of public hospitals to admit among the attending physicians persons who designate themselves and are known as advocates of partisan principles, as champions of new and special modes of practice. Thus in one of our medical colleges it is proposed to induct a Professor expressly to teach its classes the doctrine of a new school. Under these circumstances, and in a storm of indignation, the present medical staff of these hospitals are called on loudly to resign their places promptly and peremptorily; and the present highly esteemed faculty of the medical school alluded to are required to vacate their chairs, rather than accept the enforced fellowship of so equivocal a colleague. For my own part, I regard the course thus advised as full of danger, and threatening seriously evil consequences. With a single condition, which is not refused, or even contemplated to be refused, the conjoint hospital service which has been denounced as derogatory, seems to be at once safe and honorable, and indeed promises to be full of advantage. Let there be no interchange of patients; let separate wards be set apart in which each practitioner shall follow his own course, and it is impossible to see what contamination or other injury shall accrue. Nay, as we all profess to be in search of truth; as we are all anxious to provide "the greatest good for the greatest number," we must not shrink from any fair experiment, any attempt at palpable exhibition or open demonstration. Truth has nothing to fear from the frankest exposure, the most precise comparisons. I have not the slightest uneasiness as to the ultimate result. Hypocrisy and quackery may not be unmasked at once, as by the sudden touch of Ithuriel's spear; the prejudice and obstinacy of dullness and ignorance may require some time to overcome; but at last even the most partial jury will be convinced by accumulation of evidence, and the "sober second thought" of the common people, to which even science itself must not disdain to appeal, will be enlightened and guided aright.

Similar reasoning is applicable, and with greater force, in reference to the contemplated professorship. Laymen will accuse us of evading the offer of "a fair field and no favor," and of doubting our own ability to maintain what we proclaim as truth and reason. Let me be permitted to express the hope that the respected members of our profession who in New York, or Chicago, or Ann Arbor, or anywhere else, are thus, or in any similar manner, called upon and chosen "to defend the right," will show themselves as ready and willing as we know them to be able champions of our common and time-honored faith.

It is evident that Dr. Dickson here refers directly to the attempt made some months since to introduce into our new city hospital two complete, co-equal Medical Boards; one styled "Allopathic," and the other "Homœopathic." And his readers will certainly regard the doctrine he inculcates as the reverse of that which prompted me to refuse to accept an appointment under such a *title* and with such co-laborers. With the highest regard for the opinions of so distinguished a member of the professsion, I must be permitted either to differ from him on this subject, or to express the belief that he very imperfectly comprehends the questions, whether ethical or moral, which were presented by our Board of Health in their attempt to organize a medical and surgical corps for attendance on the new Chicago City Hospital.

Had our Board of Health simply proposed to set apart a ward in the Hospital, in which such patients as chose to be treated in accordance with some special *pathy* or *ism* could be admitted and gratified, very few would have objected to the arrangement. But when the Governors of an Hospital select from among the so-called exclusive or special systems of medicine, one like the Homœopathic, and appoint from among its followers a full Board of Physicians and Surgeons; and, *per contra*, assume to designate the great body of educated physicians by a title equally special and exclusive, viz: "Allopathists," and appoint from them another complete Board; and not only allow each patient to choose which department he will enter, but divide equally all such as either have no choice or are too sick to express it, they present ethical questions of a very different import. To accept places at the hands of public authorities with the specific title of "Allopathic" entered upon the public records, and under which, official reports must be made from month to month, is to acknowledge publicly, in the act itself, that the ordinary scientific practice of the day is regulated by the single narrow principle of *contraria contrariis curantur*, and is therefore as much a *pathy* as its opposite.

We hold that no enlightened physician could, consistently with truth or propriety, accept any such position. To do so, would be to aid the enemies of the profession in perpetuating popular errors, and to place one's-self positively on a level with quacks.

Again, if the Homœopathic method of treating disease, when faithfully adhered to, has been fully proved to be inefficient and unsafe when applied to diseases of an acute and severe character, is it morally or ethically right to lend our sanction as physicians to any arrangement by which the poor, who might be too sick or too ignorant to choose for themselves, should be committed irretrievably to that system? Dr. Dickson tells us that "we must not shrink from any fair experiment, any attempt at palpable exhibition or open demonstration." But suppose the questions to be investigated have already been palpably exhibited and openly demonstrated in every quarter of the globe for half a century, how long shall we consent to continue the investigation, especially when human victims are to be *involuntarily* the subjects of the experiments? When a patient voluntarily chooses to risk his health or life with the advocates of some pathy or ism, the educated physician is in no degree responsible for the result. But if the public authorities of a city open a public hospital, which is to be the only refuge of the sick poor, on such a plan that all who come unable to choose for themselves one week shall be sent into one department, and all who come the next week into another, is any intelligent physician justifiable in so far sanctioning the monstrosity as to accept a place in one of its departments? We think a proper sense of self-respect as well as every principle of sound morals will prompt an answer in the negative.

The comments of Prof. Dickson seem to be founded upon the idea that the profession ought not to oppose the appointment to public institutions those who advocate "*new* and *special* modes of practice." Without discussing the merits of the proposition itself, let us briefly inquire whether the Homœopathy of the present day is entitled to rank as either "*new*" or "*special*" in its nature? More than half a century has now elapsed since Hahnemann promulgated to the world the peculiar doctrines of Homœopathy. During that time all the physical or natural sciences auxiliary to scientific medicine have advanced beyond all previous parallel; and the application of auscultation and percussion, with other means of exploration to the diagnosis of disease, which, together with the aid of the microscope and organic chemistry in revealing the most intricate morbid changes in the fluids and solids of the human body, have almost completely metamorphosed the whole science and practice of medicine. The steam engine, the railroad locomotive, and the electric telegraph, have not made greater changes and improvements in the industrial, commercial and social world, than have been made, during the last fifty years, in the application and practice of medical science. And yet the phrases "old system of practice," "old school," etc., are daily heralded in the newspapers and sounded in the public ear by a set of men, who, under the title of a "*new school*," are blindly and dogmatically following the fanciful doctrines promulgated by a German enthusiast more than half a century ago. Homœopathy, then, is not "*new*," either in an absolute or comparative sense. Neither has the profession failed to afford it abundant opportunities for "fair experiment" and "open demonstration." On the contrary, while its progenitor was himself upon the stage of action, the claims of the pretended new system were subjected to fair and repeated trials, both in public hospitals and private practice. It often happens that the discovery of an important new fact in science, or the promulgation of a new principle or truth in philosophy, by disturbing preconceived opinions and popular prejudices, arouses against the discoverer unreasonable prejudices and opposition, which, for a time, may prevent a fair and full appreciation of such facts or truths. But all history shows that such prejudices and opposition to *real* truths are always temporary, and restricted to the generation cotemporary with the discoverer. Thus, Harvey in announcing the circulation of the blood, and Jenner the practice of vaccination, called into activity the violent opposition of many of their cotemporaries, both in and out of the profession. And yet even while they were still upon the stage of action, their discoveries became incorporated as established practical truths in the profession, and the succeeding generation began to erect monuments to their memory. And thus it ever has been, and ever will be, in regard to every real scientific truth announced to the world. How has it been with the doctrines of Homœopathy? They were first promulgated about the same time that Jenner announced the efficacy of vaccination. The latter was long since accepted and freely acknowledged as one of the greatest benefits ever conferred on the human race; while the former, though promulgated through all the literature of Christendom, discussed and experimented with more or less in almost every medical society in Europe and America, and their advocates not only allowed free access to the public mind, but at times patronized alike by priests, nobles, princes, and even crowned heads, yet remain at this time, after the lapse of fifty years, or the lifetime of nearly three generations, wholly repudiated by the profession and their pretended advocates, with scarcely a foothold or a place in a single public hospital or incorporated school of medicine on either hemisphere of our globe. And not only so, but even three-fourths of those who claim to be Homœopaths at this time, openly renounce the idea of potentizing remedies by trituration and attenuation, or the efficacy of infinitesimal doses—the very idea that gave to the system all its original novelty and interest. What then is there left, that Professor Dickson would have experimented on or more openly demonstrated?

We see but one question left in connection with Homœopathy, and that is, how long a class of men calling themselves *doctors*, can succeed in deceiving the people, by calling calomel, *mercurius;* arsenic, *arsenicum*, etc., and concealing the most concentrated and poisonous articles of the Materia Medica under a cover of sugar of milk. But Prof. Dickson and all others must excuse us from even a remote connection with such men for such a purpose.

